# Supplementary material for: Health Literacy is Associated with Health Behaviors in Students from Vocational Education and Training Schools: A Danish Population-Based Survey
Source: Int J Environ Res Public Health. 2020 Jan 20;17(2):671. doi: 10.3390/ijerph17020671 (PMC7014204; doi:10.3390/ijerph17020671)
Supplement: Supplementary file 1 [file ijerph-17-00671-s001.pdf]

International Journal of
Environmental Research and Public Health

MDPI

Supplementary Materials S1. Table S1

Table S1: Associations between health literacy domains and health behavior among VET-school students 19+ years old.

	Scale 3. Managing health				Scale 5. Appraisal of health information			
	Crude		Adjusted*		Crude		Adjusted*	
	OR	(95% CI)	OR	(95% CI)	OR	(95% CI)	OR	(95% CI)
**Dietary habits**								
Every day (ref)	1		1		1		1	
Somedays	**0.604**	(0.530-0.687)	**0.666**	(0.582-0.762)	**0.753**	(0.663-0.855)	**0.833**	(0.729-0.951)
Seldom or never	**0.482**	(0.422-0.550)	**0.548**	(0.478-0.629)	**0.624**	(0.549-0.710)	**0.700**	(0.612-0.801)
**Smoking status**								
Never (ref)								
Former	0.970	(0.846-1.113)	0.955	(0.826-1.104)	*0.879*	(0.759-1.019)	*0.876*	(0.759-1.011)
Occasional	0.877	(0.703-1.095)	0.910	(0.718-1.154)	*0.821*	(0.657-1.026)	0.868	(0.686-1.098)
Daily	**0.613**	(0.531-0.708)	**0.666**	(0.572-0.775)	**0.736**	(0.639-0.847)	**0.794**	(0.686-0.918)
**Alchohol**								
No-low risk (ref)								
Intermediate risk	*0.844*	(0.701-1.016)	0.907	(0.745-1.103)	**0.770**	(0.639-0.927)	*0.843*	(0.695-1.022)
High risk	**0.641**	(0.543-0.758)	**0.759**	(0.633-0.909)	**0.689**	(0.583-0.816)	**0.831**	(0.695-0.993)
**Physical activity**								
High (ref)								
Moderate	**0.724**	(0.623-0.841)	**0.702**	(0.598-0.825)	*0.879*	(0.759-1.019)	**0.841**	(0.720-0.983)
Low	**0.396**	(0.345-0.455)	**0.430**	(0.372-0.498)	**0.611**	(0.537-0.695)	**0.642**	(0.560-0.736)

Adjusted for gender, age, educational level, main educational area, self-rated health, BMI

Bold: $p<0.05$, Italic: $p<0.1$
